# Dependency‐Distance Effects Are Not Evidence Against Grammatical Theories With Empty Categories: When Psycholinguistic Data Are Not Diagnostic

**DOI:** 10.1111/cogs.70255

**Published:** 2026-08-19

**Authors:** Hiroki Fujita

**Affiliations:** ^1^ Department of Linguistics University of Potsdam

**Keywords:** Sentence processing, Parsing, Syntax, Empty category, Dependency, Language comprehension

## Abstract

A long‐standing question in cognitive science is whether psycholinguistic evidence can adjudicate among grammatical theories. This question arises in debates over the representation of linguistic dependencies: whether they are mediated by unpronounced elements (empty categories; ECs) or established without them. Some psycholinguistic research has taken dependency‐distance effects—the observation that longer dependencies impose greater processing cost—as evidence in favor of EC‐free over EC‐mediated accounts, and this claim has recently been revived. In this letter, I argue that this conclusion is unwarranted. Once the incremental, real‐time nature of sentence processing is taken into account, dependency‐distance effects are expected under EC‐mediated accounts as well. I show how EC‐mediated accounts can explain these effects and review findings consistent with this view. Dependency‐distance effects are, therefore, not diagnostic of whether dependencies involve ECs. More broadly, psycholinguistic data can be misleading when used to draw inferences about grammatical theories without reference to the real‐time, incremental processes that give rise to those data.

## 1 Introduction

In cognitive science, the relationship between grammar and parsing has long been debated (e.g., Berwick & Weinberg, 1984; J. Fodor, A., T., & M., 1974; Kush & Dillon, 2023; Marantz, 2005; Miller & Chomsky, 1963; Phillips, 1996; Phillips & Lau, 2004; Phillips & Lewis, 2013; Sprouse & Lau, 2013; Trotzke, Bader, & Frazier, 2013). A central question in this debate is whether psycholinguistic evidence can adjudicate among competing grammatical theories. One case in which this question arises concerns how dependencies—grammatical relationships between two elements—are mentally represented. To illustrate, consider (1).


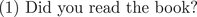


This sentence contains the verb “*read*,” which takes the noun phrase “*the book*” as its object. It is uncontroversial that “*read*” and “*the book*” form a local verb–object dependency in the underlying sentence structure.

Consider now (2).


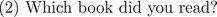


In (2), “*Which book*” appears at the beginning of the sentence. There is no dispute that this wh‐phrase forms a verb–object dependency with “*read*.” However, controversy arises over how this dependency is represented in the underlying sentence structure.

On one account, “*read*” always enters into a local relation with its object noun phrase, but that noun phrase may surface elsewhere in the sentence, subject to certain constraints (e.g., Berwick & Weinberg, 1984; Chomsky, 1977, 1981; J. D. Fodor, 1978). On this empty‐category‐mediated (EC‐mediated) account, the local relation between “*Which book*” and “*read*” is established via a representation of “*Which book*” in the object position of “*read*.” This representation is silent in English, but in some other languages (e.g., Hebrew, Irish), it can be realized as a pronoun under certain conditions (e.g., Ackerman, Frazier, & Yoshida, 2018; Borer, 1984; McCloskey, 2017; Meltzer‐Asscher, 2021; Morgan & Wagers, 2018; Sells, 1987). Such silent representations are often referred to as empty categories.

On another account, “*read*” can stand in either a local or a nonlocal relation to its object noun phrase (e.g., da Cunha & Gibson, 2025; Gazdar, Klein, Pullum, & Sag, 1984; Kaplan & Zaenen, 1989; Sag & Fodor, 1994). Under this EC‐free account, “*read*” in (2) forms a nonlocal dependency with “*Which book*,” without the mediation of an EC. Simplified variants of the EC‐mediated and EC‐free accounts are illustrated below.


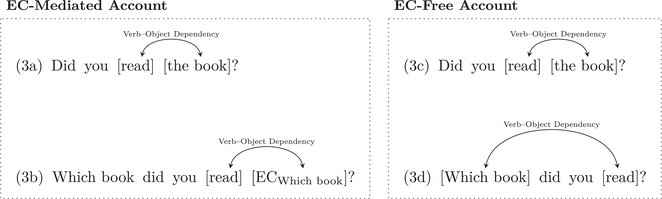


EC‐mediated and EC‐free accounts posit distinct mechanisms to capture the configurations in (1) and (2). Both mechanisms are conceptually simple, and determining which of the two accounts is correct has long posed a challenge for linguists. This has led some psycholinguists to ask whether psycholinguistic data can help adjudicate between them. Pickering and Barry (1991) offered an early argument along these lines, and da Cunha and Gibson (2025) recently revived it. The core reasoning is as follows:


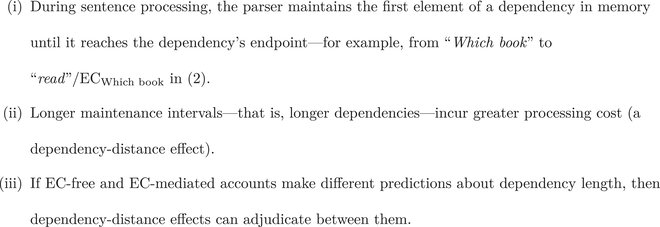


Following this line of reasoning, Pickering and Barry (1991) and da Cunha and Gibson (2025) examined particular dependencies and concluded that dependency‐distance effects uniquely support EC‐free accounts.

In this letter, I argue that this conclusion is unwarranted. Dependency‐distance effects can be explained under EC‐mediated accounts as well and, therefore, do not adjudicate between EC‐mediated and EC‐free accounts. In the following sections, I first describe dependency‐distance effects, then review previous research supporting this argument, and finally show how EC‐mediated accounts can explain them.

## 2 Dependency‐distance effects

To illustrate dependency‐distance effects, consider the experimental design used by da Cunha and Gibson (2025):


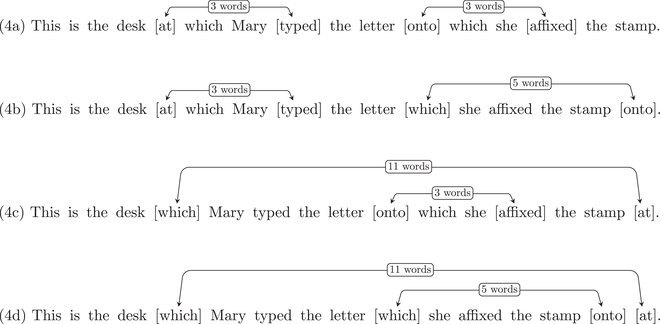


In (4a–d), the arcs represent the critical dependency length. As the labels show, dependency length increases progressively from (4a) to (4d). Accordingly, this account predicts a corresponding increase in processing cost (PC): PC_(4a)_ < PC_(4b)_ < PC_(4c)_ < PC_(4d)_.

Now consider the same sentences under da Cunha and Gibson's EC‐mediated account and distance metric, focusing on the dependencies between the wh‐phrases and their ECs:


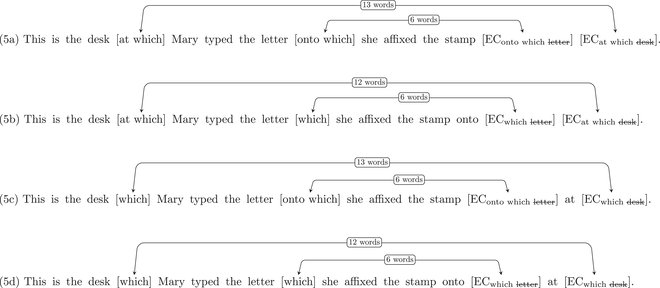


In (5a–d), wh–EC dependency lengths are largely matched across conditions (for a more formal description of the syntactic structures, see the Appendix). On da Cunha and Gibson's reasoning, their EC‐mediated account, therefore, predicts similar processing costs across (5a–d). They tested this prediction in an acceptability judgment task, assuming that higher processing costs reduce acceptability, and found a graded decrease in ratings (R) across conditions: R_(4a/5a)_ = 4.07, R_(4b/5b)_ = 3.87, R_(4c/5c)_ = 3.22, R_(4d/5d)_ = 3.01.

da Cunha and Gibson (2025) claim that this pattern is more consistent with EC‐free accounts and conclude that dependencies are formed without ECs. However, this conclusion is unwarranted. This is because syntactic structure building does not proceed in lockstep with surface word order, and the relevant dependencies are formed during real‐time processing at points that do not coincide with their endpoints in the sentence as a whole. Once this aspect of real‐time sentence processing is taken into account, da Cunha and Gibson's findings are also compatible with EC‐mediated accounts.

A similar objection was raised by Gibson and Hickok (1993) in response to Pickering and Barry (1991), who drew the same conclusion as da Cunha and Gibson (2025). da Cunha and Gibson dismiss this objection on the grounds that previous research does not provide evidence that uniquely supports an EC‐mediated analysis of the relevant dependencies. However, whether there is such evidence is beside the point here. Rather, the relevant question is whether there is empirical evidence compatible with an EC‐mediated explanation of dependency‐distance effects.

Since Gibson and Hickok (1993), a growing body of research has shown that the parser predicts dependency endpoints and forms dependencies before those endpoints are confirmed during real‐time processing. This line of research provides such evidence. In the following sections, I review this research and show how EC‐mediated accounts can explain da Cunha and Gibson's (2025) results.

## 3 Parsing and dependency formation in real‐time processing

Substantial evidence suggests that dependencies are formed at the earliest possible opportunity, before their endpoints are confirmed.

For example, consider (6), in which the ultimately grammatically licensed position for the EC associated with “*Which brush*” is immediately after “*with*,” but an EC can be temporarily posited earlier at “*clean*.”


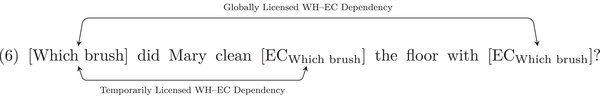


A large body of research indicates that processing difficulty arises at “*the floor*” (e.g., Crain & Fodor, 1985; Fujita & Cunnings, 2020; Pickering & Barton, 1994; Stowe, 1986; Wagers & Phillips, 2014; see also Fujita & Cunnings, 2022; Pickering & Traxler, 2003; Traxler & Pickering, 1996; Wagers & Phillips, 2009, for related findings). These findings suggest that the parser temporarily forms a verb–object dependency at “*clean*” before confirming the ultimately licensed EC position:


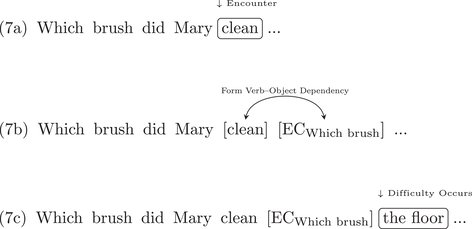


Such processes—linking fronted wh‐phrases to the linearly closest verbs—have also been observed in Japanese, a head‐final language, even when other verbs, though linearly more distant, are structurally closer to the wh‐phrases (e.g., Aoshima, Phillips, & Weinberg, 2004; Omaki, Davidson White, Goro, Lidz, & Phillips, 2014; see also Chacón et al., 2016, for similar/related findings in Bangla).

Research further suggests that ECs are posited predictively (e.g., Aoshima et al., 2004; Omaki et al., 2015):


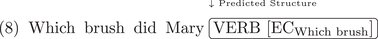


In (8), a verb phrase containing an EC is predicted. Such predictive processes are also compatible with previous research showing that large amounts of syntactic structure can be predicted (e.g., Fujita, 2023; Kush, Lidz, & Phillips, 2017; Phillips, 2006; Yoshida, 2006) and that the licensing of an EC (or, in some languages, a pronoun) can be determined before it appears (e.g., Keshev & Meltzer‐Asscher, 2017; Omaki & Schulz, 2011; Stowe, 1986; Traxler & Pickering, 1996).

These studies show that the point at which a dependency is recognized and formed during real‐time processing does not necessarily coincide with its endpoint in the sentence as a whole. This suggests that da Cunha and Gibson's (2025) data do not straightforwardly adjudicate between EC‐mediated and EC‐free accounts.

## 4 EC‐mediated accounts of dependency‐distance effects

In this section, I show how EC‐mediated accounts can explain the dependency‐distance effects in (5a–d) in light of the research reviewed in Section 3. There are two possible approaches: one that focuses on bottom‐up processes and another that posits top‐down processes.

First, consider the bottom‐up account. As discussed in Section 3, previous research suggests that dependencies are formed at the earliest possible opportunity. In (5a/b), for the first prepositional wh‐phrase (“*at which*”), the earliest point at which an EC can be posited is immediately after “*typed (the letter)*”:


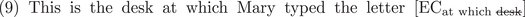


If the EC is posited at this point, a verb–location dependency is established (10a), and the subsequent words can be processed without disrupting this dependency (10b)^1^:


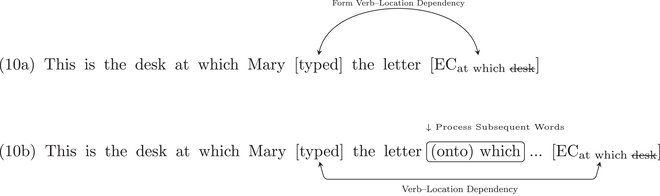


Thus, once “*typed (the letter)*” has been encountered, the wh‐phrase no longer needs to be maintained in memory, resulting in a relatively low processing cost.

Now consider (5c/d), focusing on the first nonprepositional wh‐phrase (“*which*”). In these cases, no plausible EC position becomes available until the sentence‐final preposition “*at*” is encountered (11a):


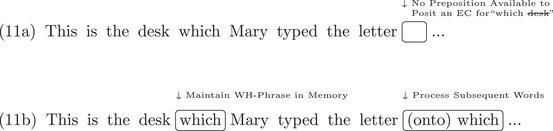


As a result, the wh‐phrase is maintained in memory over a longer span (11b), leading to a relatively high processing cost. Since the number of nonprepositional wh‐phrases increases progressively from (5a) to (5d), the bottom‐up account can explain the graded decrease in acceptability observed by da Cunha and Gibson (2025).

Next, consider the top‐down account. As discussed in Section 3, previous research suggests that ECs can be posited predictively during real‐time processing. Following this research, suppose that, in (5a/b), by the time “*Mary*” is processed, an EC has been posited:


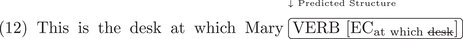


When “*typed (the letter)*” is encountered, a dependency between “*typed*” and the predicted EC is formed:


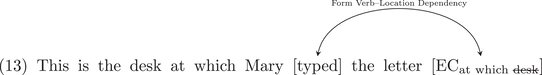


At this point, the wh‐phrase no longer needs to be maintained in memory, resulting in a relatively low processing cost.

In (5c/d), an EC is predicted in a similar way:


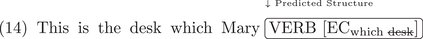


However, when the subsequent words “*typed the letter (onto) which*” are encountered, the preposition “*at*”—which is necessary to connect the EC with “*typed*”—has not yet appeared:


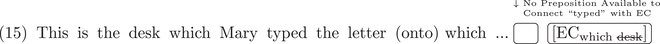


As a result, the wh‐phrase continues to be maintained in memory, leading to a relatively high processing cost.

Taken together, these analyses show that the acceptability pattern reported by da Cunha and Gibson (2025) can be explained under EC‐mediated accounts.

Finally, factors other than dependency length may also have contributed to the observed acceptability pattern. For example, in the nonprepositional wh‐phrase condition (“*This is the desk which Mary typed the letter…*”), previous research suggests that “*desk*” is initially analyzed, via an EC, as the direct object of “*typed*.” This analysis is expected to cause processing difficulty because “*desk*” is a semantically implausible object of “*typed*.” No such difficulty arises in the prepositional wh‐phrase condition (“*This is the desk at which Mary typed the letter…*”). Although the present paper has focused on dependency length, all processing‐related factors should be taken into account when drawing inferences about grammatical theories from psycholinguistic data.

## 5 Conclusion

In this letter, I argued that the dependency‐distance effects reported by da Cunha and Gibson (2025) can be explained under EC‐mediated accounts once the real‐time, incremental nature of sentence processing is taken into account. These effects, therefore, do not uniquely support EC‐free accounts and are not diagnostic of whether dependencies involve ECs. More broadly, the argument developed here suggests that psycholinguistic data can be misleading when used to draw inferences about grammatical theories without reference to the real‐time, incremental processes that give rise to those data.

Although the present discussion is situated within linguistics, the broader methodological lesson extends across cognitive science: data shaped by dynamic processing do not necessarily warrant straightforward inferences about mental structure. Such inferences are best justified when evaluated in light of how the relevant processes unfold over time and which factors shape those processes.

## Funding

This study was supported by the Deutsche Forschungsgemeinschaft (German Research Foundation)—project number 500540359; primary investigator: Hiroki Fujita.

## Conflict of Interest Statement

The author has no relevant financial or nonfinancial interests to disclose.

## Ethics approval statement

No ethical approval was required because this study did not involve human participants.

## Permission to reproduce material from other sources

This manuscript does not include any material reproduced from other sources.

## Figures and Tables

**Fig. A1 cogs70255-fig-0001:**
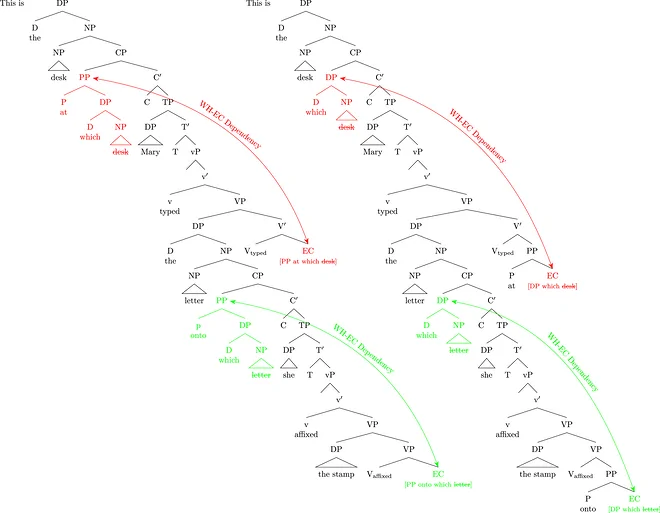
Formal description of the syntactic structures in (5a) and (5d).

## Data Availability

This study does not involve any data.

## Note

1 See the Appendix for an illustration of how this dependency is locally represented in the underlying sentence structure.

## 1

[Fig cogs70255-fig-0001]
